# Encoding of menstrual pain experience with theta oscillations in women with primary dysmenorrhea

**DOI:** 10.1038/s41598-017-16039-4

**Published:** 2017-11-22

**Authors:** Pin-Shiuan Lee, Intan Low, Yong-Sheng Chen, Cheng-Hao Tu, Hsiang-Tai Chao, Jen-Chuen Hsieh, Li-Fen Chen

**Affiliations:** 1 Institute of Biomedical Informatics, National Yang-Ming University, Taipei, Taiwan; 2 Department of Computer Science, National Chiao Tung University, Hsinchu, Taiwan; 3 Integrated Brain Research Unit, Division of Clinical Research, Department of Medical Research, Taipei Veterans General Hospital, Taipei, Taiwan; 4 Department of Obstetrics and Gynecology, Taipei Veterans General Hospital, Taipei, Taiwan; 5 Institute of Brain Science, National Yang-Ming University, Taipei, Taiwan

## Abstract

Theta oscillation (4–7 Hz) is well documented for its association with neural processes of memory. Pronounced increase of theta activity is commonly observed in patients with chronic neurogenic pain. However, its association with encoding of pain experience in patients with chronic pain is still unclear. The goal of the present study is to investigate the theta encoding of sensory and emotional information of long-term menstrual pain in women with primary dysmenorrhea (PDM). Forty-six young women with PDM and 46 age-matched control subjects underwent resting-state magnetoencephalography study during menstrual and periovulatory phases. Our results revealed increased theta activity in brain regions of pain processing in women with PDM, including the right parahippocampal gyrus, right posterior insula, and left anterior/middle cingulate gyrus during the menstrual phase and the left anterior insula and the left middle/inferior temporal gyrus during the periovulatory phase. The correlations between theta activity and the psychological measures pertaining to pain experience (depression, state anxiety, and pain rating index) implicate the role of theta oscillations in emotional and sensory processing of pain. The present study provides evidence for the role of theta oscillations in encoding the immediate and sustained effects of pain experience in young women with PDM.

## Introduction

Pain engages integrative processes in various regions of the brain, including those associated with the limbic system^1^. As a form of stress, chronic pain is often accompanied by co-morbid affective symptoms^2^, such as depression and anxiety^3^. Long-term pain can cause neurophysiological and psychological changes^1^ associated with neuronal oscillations at various frequencies^4^. Recent neuroimaging findings have indicated that corticolimbic circuitry characterized by altered frequency spectrum are causally involved in the development and maintenance of chronic pain^5,6^. An increase in theta oscillations is the most common abnormality in patients with chronic pain^4^. Previous electroencephalographic (EEG) studies have reported a shift in the dominant frequency to the theta band in patients with neuropathic pain^7,8^. Theta oscillations with abnormal amplitudes have also been observed in cases involving chronic neuropsychiatric disorders^9^. However, researchers have yet to elucidate the role of theta oscillations in mediating the affective symptoms in the experience of pain in patients with chronic pain.

Primary dysmenorrhea (PDM) is a form of chronic pelvic pain associated with menstruation but without an identifiable pelvic pathology^10,11^. The cyclic nature of PDM makes it an ideal model by which to unravel the neural mechanisms underlying the experience of spontaneous pain (menstrual phase) and the stress associated with the anticipation of forthcoming events during pain-free periods (periovulatory phase). In a series of our PDM studies, we reported functional or structural changes in several pain-related regions of the brain that can be indicative of adaptive or maladaptive neuroplasticity^12–14^. We also found functional connectivity between the ventromedial prefrontal cortex (vmPFC) and anterior insula (aINS) as well as the dorsal anterior cingulate cortex (dACC), with shifts from positive to negative correlation across the menstrual cycle, suggesting that pain-primed anxiety could be adapted^12^.

It has been reported that the insula and cingulate cortex are associated with the experience of anxiety^8,15–17^ and interoceptive awareness^18^. The functional significance of connectivity between specific subdivisions of these two areas in a resting state has also been reported^19^. One system operating between the aINS and posterior ACC (pACC) in conjunction with the anterior part of middle cingulate cortex (aMCC) may be involved in the integration of interoceptive information with emotional salience to form a subjective representation of the body. The other system operating between the entire insula and posterior MCC (pMCC) would be associated with general salience, response selection, and action. Due to the prevalence of anxiety in women with PDM^12,15^, we hypothesized that the insula and cingulate cortex may play important roles in encoding the experience of pain and its sustained emotional stress throughout the menstrual cycle.

Based on the adaptive control hypothesis, researchers have posited that anxiety-related theta oscillations in MCC may be associated with cognitive control^16^. Researchers have also observed elevated frontal-midline theta signals in response to adverse stimuli, such as pain^16^. In a recent PDM study based on resting MEG data, we demonstrated the importance of the low frequency component in the prediction of pain intensity in cases of spontaneous pain^17^. In the present study, we further examined the role of theta oscillations in affective symptoms of pain experience and in women with PDM, particularly in the insula and cingulate cortex. We hypothesized that theta oscillations of PDM subjects would be increased in pain-related regions (including somatosensory cortex, thalamus, insula, and cingulate cortex)^20,21^ under the effects of pain, and that these altered theta oscillation would be associated with anxiety during pain-free stages.

## Results

### Demographic and psychological data

Table 1 lists the demographic characteristics of the 46 PDM subjects and 46 healthy controls (CON) enrolled in this study. We observed no significant between-group differences with regard to age, age at menarche, years of menstruation, average duration of the menstrual cycle, or handedness. PDM subjects averaged 9.12 ± 2.95 years of pain, 63% reported absences from school or work, and 45.7% reported the use of analgesics. The overall experience of pain was assessed using the total Pain Rating Index (PRI) from the McGill Pain Questionnaire (MPQ) (MENS, 34.35 ± 14.03; POV, 35.98 ± 13.06). Our PRI findings confirm that the PDM subjects experienced moderate to severe menstrual pain. The Short-Form Health Survey (SF-36) for measuring the quality of life revealed that PDM subjects exhibited mental well-being (MENS, 46.84 ± 11.29; POV, 46.21 ± 11.19) and physical well-being (MENS, 48.06 ± 8.49; POV, 47.52 ± 8.50) lower than that of CON (mental: MENS, 55.52 ± 7.05; POV, 56.42 ± 6.85; physical: MENS, 55.42 ± 3.06; POV, 55.08 ± 3.61), regardless of whether the subject was in the MENS phase (p < 0.0001) or POV phase (p < 0.0001) (Table 1).

**Table 1:** Demographic data and baseline information.

	MENS phase		POV phase			
	PDM (n = 46)	CON (n = 46)	*P*	PDM (n = 46)	CON (n = 46)	*P*
Age, y	23.46 ± 2.38	24.09 ± 2.67	0.268	23.07 ± 2.06	23.54 ± 2.39	0.359
Age at menarche	12.02 ± 1.05	12.20 ± 1.14	0.474	12.11 ± 1.12	12.34 ± 1.06	0.293
Years of menstruating	11.37 ± 2.60	11.89 ± 2.93	0.420	10.97 ± 2.45	11.21 ± 2.59	0.626
Days of 1 menstrual cycle	29.48 ± 1.32	29.49 ± 1.06	0.997	29.53 ± 1.37	29.66 ± 1.05	0.630
Menstrual pain experience						
Pain history, y	9.12 ± 2.95	—	—	8.87 ± 2.74	—	—
Absenteeism, %	63.0	—	—	67.4	—	—
Drug taken, %	45.7	—	—	45.7	—	—
Recalled PRI scores	34.35 ± 14.03	—	—	35.98 ± 13.06	—	—
Present PRI scores (range, 0–78)	29.61 ± 11.65	—	—	—	—	—
Sensory (range, 0–42)	16.00 ± 5.67	—	—	—	—	—
Affective (range, 0–14)	3.95 ± 2.75	—	—	—	—	—
Evaluation (range, 0–5)	2.39 ± 2.04	—	—	—	—	—
Miscellaneous (range, 0–17)	7.27 ± 3.77	—	—	—	—	—
Present PPI scores (range, 0–5)	2.73 ± 1.05	—	—	—	—	—
Edinburgh Handedness Inventory	83.66 ± 16.61	83.53 ± 20.84	0.705	82.99 ± 16.94	85.34 ± 16.20	0.396
SF-36						
Mental component summary	46.84 ± 11.29	55.52 ± 7.05	0.001*	46.21 ± 11.19	56.42 ± 6.85	<0.0001*
Physical component summary	48.06 ± 8.49	55.42 ± 3.06	<0.0001*	47.52 ± 8.50	55.08 ± 3.61	<0.0001*
Total value	94.90 ± 13.44	111.11 ± 6.64	<0.0001*	93.73 ± 13.76	111.50 ± 6.45	<0.0001*

*Denotes a significant between-group difference at *p* < 0.05 with Bonferroni correction. MENS, menstruation; POV, periovulatory; PDM, primary dysmenorrhea; CON, healthy controls; PRI, pain rating index; PPI, present pain intensity; SF-36, Short Form Health Survey.

Table 2 lists the psychological data of the participants. In the psychological assessments, we observed significant group differences in state anxiety (MENS, *p* < 0.0001; POV, *p* = 0.0002) and trait anxiety (MENS, *p* < 0.0001; POV, *p* < 0.0001) using the Spielberger State-Trait Anxiety Inventory (STAI), Beck Depression Inventory (BDI) (MENS, *p* < 0.0001; POV, *p* = 0.023), Beck Anxiety Inventory (BAI) (MENS, *p* < 0.0001; POV, *p* < 0.0001), and Pain Catastrophizing Scale (PCS) (MENS, *p* < 0.0001; POV, *p* < 0.0001). The pain experience during MENS phase, as assessed by the total present PRI scores from MPQ (29.61 ± 11.65), confirmed that PDM subjects experienced moderate menstrual pain.

**Table 2:** Results of psychological and behavioral assessments.

	PDM (n = 46)	CON (n = 46)	*P*
STAI-state (range, 20–80)			
MENS phase	42.96 ± 9.47	33.74 ± 6.38	<0.0001*
POV phase	37.13 ± 7.57	31.80 ± 6.10	0.0002*
STAI-trait (range, 20–80)			
MENS phase	45.24 ± 8.69	38.44 ± 6.74	<0.0001*
POV phase	44.84 ± 8.70	36.74 ± 6.51	<0.0001*
BDI (range, 0–63)			
MENS phase	12.26 ± 9.76	5.09 ± 4.57	<0.0001*
POV phase	7.30 ± 8.32	3.96 ± 5.17	0.023
BAI (range, 0–63)			
MENS phase	12.46 ± 8.51	2.91 ± 2.96	<0.0001*
POV phase	7.74 ± 6.29	3.07 ± 3.00	<0.0001*
PCS total scores (range, 0–52)			
MENS phase	21.28 ± 12.68	6.85 ± 7.25	<0.0001*
POV phase	19.78 ± 11.41	6.18 ± 7.65	<0.0001*
Pain rumination (range, 0–16)			
MENS phase	7.93 ± 4.75	2.80 ± 3.01	<0.0001*
POV phase	7.74 ± 4.49	2.38 ± 3.09	<0.0001*
Pain helplessness (range, 0–24)			
MENS phase	9.85 ± 6.21	2.80 ± 3.32	<0.0001*
POV phase	8.63 ± 5.46	2.58 ± 3.51	<0.0001*
Pain magnification (range, 0–12)			
MENS phase	3.50 ± 2.68	1.24 ± 1.72	<0.0001*
POV phase	3.41 ± 2.65	1.22 ± 1.72	<0.0001*

*Denotes a significant between-group difference at *p* < 0.05 with Bonferroni correction. PDM, primary dysmenorrhea; CON, healthy controls; MENS, menstruation; POV, periovulatory; STAI, Spielberger State-Trait Anxiety Inventory; BDI, Beck Depression Inventory; BAI, Beck Anxiety Inventory; PCS, Pain Catastrophizing Scale.

### MEG data

**Figure 1:**
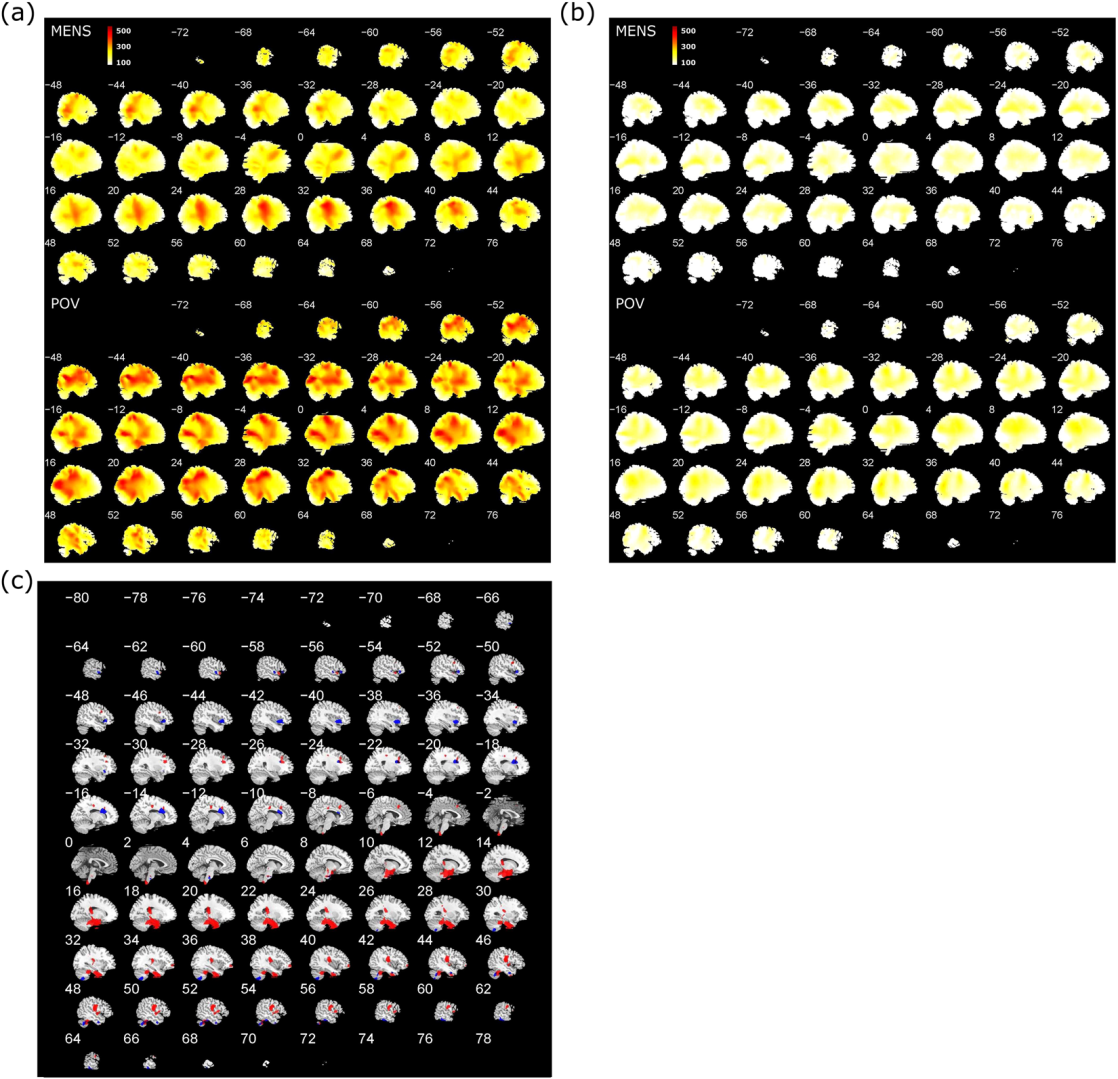
Distribution maps of theta activity in both MENS and POV phases. (**a**) Theta activation map of one representative PDM subject. (**b**) Theta activation map of one representative CON subject. Color of each voxel denotes the activation index of theta activity. (**c**) Distribution maps of increased theta activity in PDM group compared to CON group (uncorrected p < 0.01, cluster size k > 50), superimposed on T1-weighted template images. Brain regions revealed in our results (red: MENS, blue: POV) include the parahippocampal gyrus, insula, sensorimotor cortex, middle cingulate cortex, and prefrontal cortex. PDM, primary dysmenorrhea; CON, healthy controls; MENS, menstruation; POV, periovulatory.

Figure 1(a–c) present the cortical theta activity of a representative PDM subject, that of a representative CON subject, and the comparison results of two-sample t-tests between PDM and CON groups, respectively. The significance level was set at the uncorrected voxel-level p < 0.01 with cluster size > 50. The PDM group presented elevated cortical theta activity in both phases as well as increased theta activity in the painful state (MENS phase) in brain regions involved in sensory-discriminative, affective-motivational, and cognitive-evaluative processes, including the posterior insula (pINS), bilateral MCC, bilateral primary somatosensory area (SI), right secondary somatosensory area (SII), parahippocampal gyrus, and limbic areas. These findings are in line with previous studies on evoked-pain. During the pain-free state (POV phase), PDM subjects displayed increased theta activity in the left anterior insula, bilateral MCC, left SI/SII, left limbic areas, and bilateral prefrontal cortex. Notably, the left anterior and posterior parts of MCC and dorsolateral prefrontal cortex exhibited increased theta activity in both phases.

Table 3 lists the brain regions identified in statistical analysis of the data, using an uncorrected voxel-level threshold of p < 0.01 with cluster size k > 50 voxels, followed by a cluster-level permutation-test p < 0.05 (10,000 permutations). To further examine the statistical power of our results, we calculated the standardized effect sizes using Cohen’s d^22^ and the results were 0.93 (large) for PDM vs. CON during MENS phase, 0.61 (medium) for PDM vs. CON during POV phase, and 0.45 (medium) for MENS vs. POV for PDM group. It was observed that during the painful state (MENS phase), PDM subjects exhibited theta activity higher than that of healthy controls in the right parahippocampal gyrus, right pINS, left middle frontal gyrus/aMCC, and pMCC. During the pain-free state (POV phase), the PDM subjects displayed higher theta activity in the left aINS, left middle temporal gyrus, and right inferior temporal gyrus. As for the between-phase planned contrast, only in the left pre/post-central gyrus was theta activity in the PDM group higher during the POV phase than during the MENS phase.

**Table 3:** Brain regions showing between-group differences and within-group differences in theta activity.

Contrast	Anatomical label	BA	Peak coordinate (mm)			Cluster size	*t*-Score	*p*-Value
			x	y	z			
Between-group planned contrast								
MENS								
PDM > CON	Right parahippocampal gyrus	20	16	−4	−34	548	3.35	0.0006*
	Right posterior insula	43	48	−16	20	28	3.22	0.0009*
	Left middle frontal gyrus/anterior middle cingulate gyrus	32/8	−26	28	24	213	2.70	0.0042*
	Left posterior middle cingulate gyrus	24	−12	−18	40	87	2.61	0.0053*
POV								
PDM > CON	Left anterior insula	47	−40	16	−8	98	2.88	0.00247*
	Left middle temporal gyrus	21	−64	0	−6	111	2.74	0.00367*
	Right inferior temporal gyrus	20	50	−4	−34	79	2.64	0.00483*
Between-phase planned contrast								
PDM								
MENS < POV	Left pre/post-central gyrus	6/43	−60	−2	22	255	3.32	0.0009*

Peak coordinates refer to the Montreal Neurological Institute (MNI) space. Significance was thresholded at the uncorrected voxel level p < 0.01 with cluster size k > 50 voxels followed by the permutation-test cluster level p < 0.05 (denoted as*). BA, Brodmann area; PDM, primary dysmenorrhea; CON, healthy controls; MENS, menstruation; POV, periovulatory.

### Correlation results

**Figure 2:**
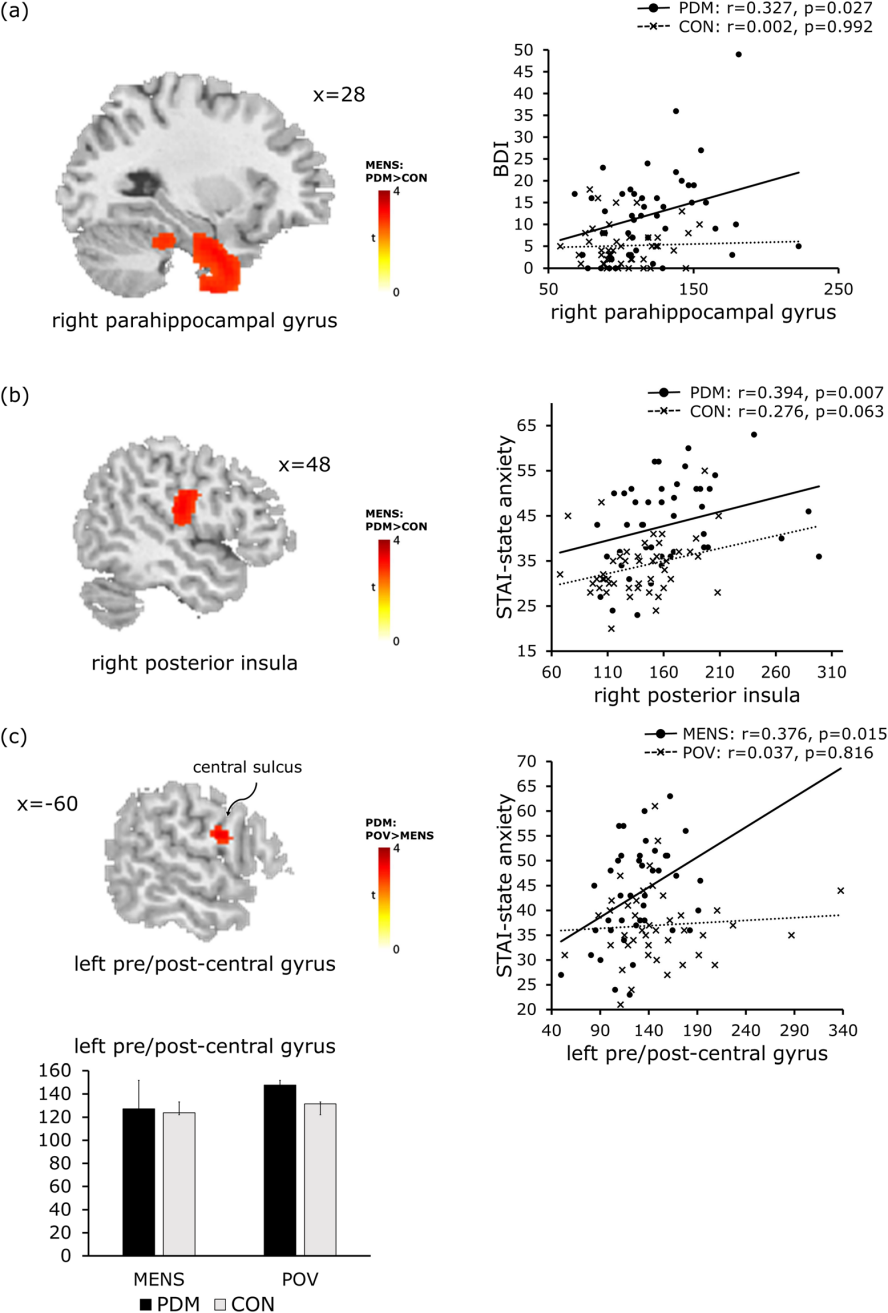
Correlation between cortical theta activity and anxiety/depression in PDM group during painful state (MENS). The left panels of (**a**), (**b**) and (**c**) illustrate the brain regions showing an increase in theta activity, as derived from between-group comparisons and between-phase comparisons, respectively (as listed in Table 3). Theta activity of the right parahippocampal gyrus is positively correlated with BDI (right panel in (**a**)). Theta activity in the right posterior insula (right panel in (**b**)) and the left pre/post-central gyrus (right panel in (**c**)) is positively correlated with the state anxiety score. MENS, menstruation; PDM, primary dysmenorrhea; CON, healthy controls; BDI, Beck Depression Inventory; STAI, Spielberger State-Trait Anxiety Inventory.

Our data revealed a positive correlation between cortical theta activity and psychological status in the painful state (MENS), but not in the pain-free state (POV). Theta activity in the right parahippocampal gyrus was correlated with depression scores in the PDM group (*r* = 0.327, *p* = 0.027), but not in the CON group (*r* = 0.02, *p* = 0.992) (Fig. 2a). The scores of state anxiety in the PDM group were significantly correlated with theta activity in the right pINS (*r* = 0.394, *p* = 0.007; Fig. 2b) and left pre/post-central gyrus (*r* = 0.376, *p* = 0.015; Fig. 2c). The CON group displayed a weak correlation between state anxiety and theta activity in the right pINS (*r* = 0.276, *p* = 0.063; Fig. 2b).

**Figure 3:**
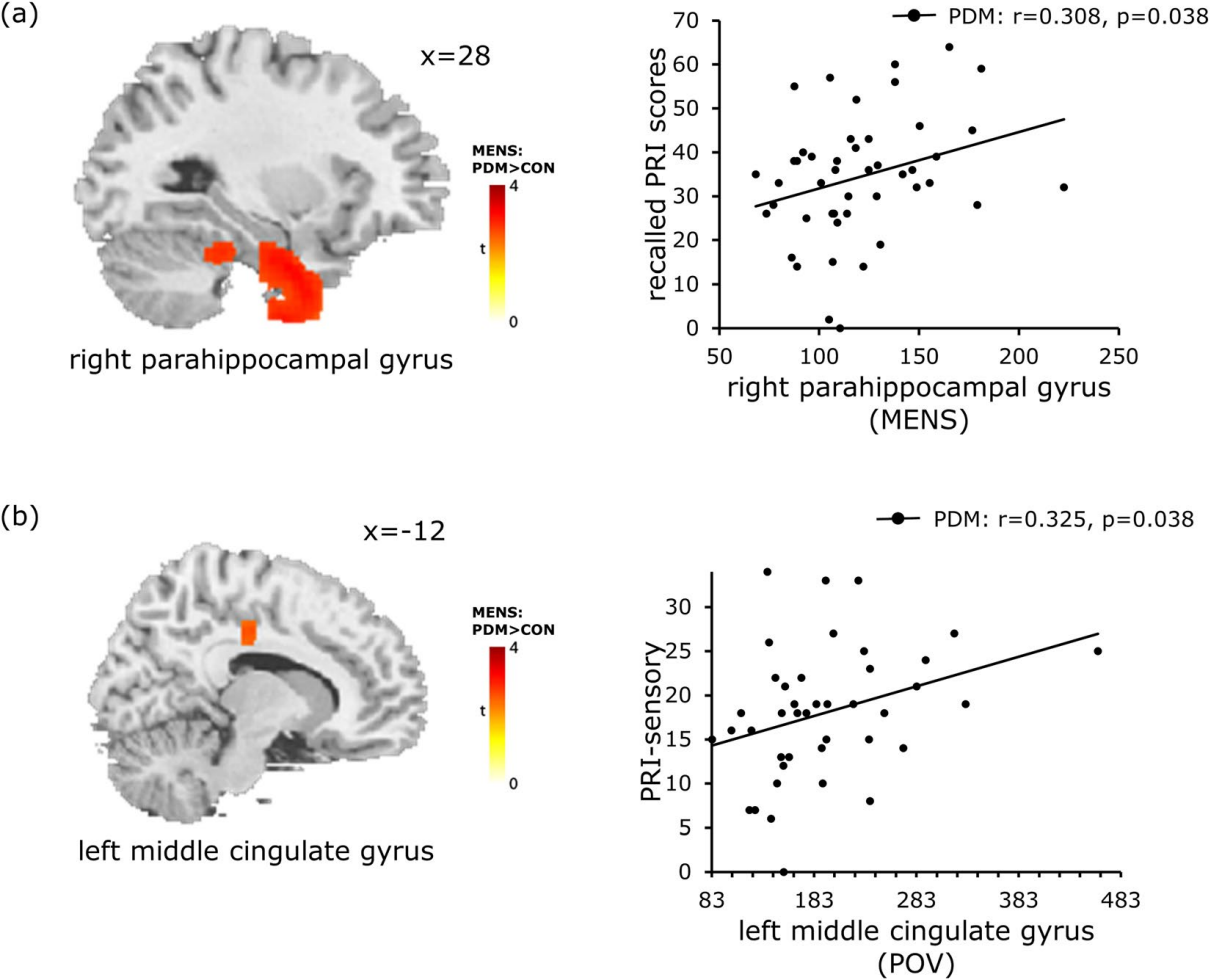
Correlation between cortical theta activity and pain experience in PDM group. The left panels of (**a**) and (**b**) illustrate the brain regions showing an increase in theta activity, as derived from between-group comparisons (as listed in Table 3). The pain rating scores are positively correlated with theta activity in the right parahippocampal gyrus during the painful state (MENS) (right panel in (**a**)) and at the left middle cingulate gyrus during the pain-free state (POV) (right panel in (**b**)). PDM, primary dysmenorrhea; CON, healthy controls; MENS, menstruation; POV, periovulatory; PRI, pain rating index.

**Figure 4:**
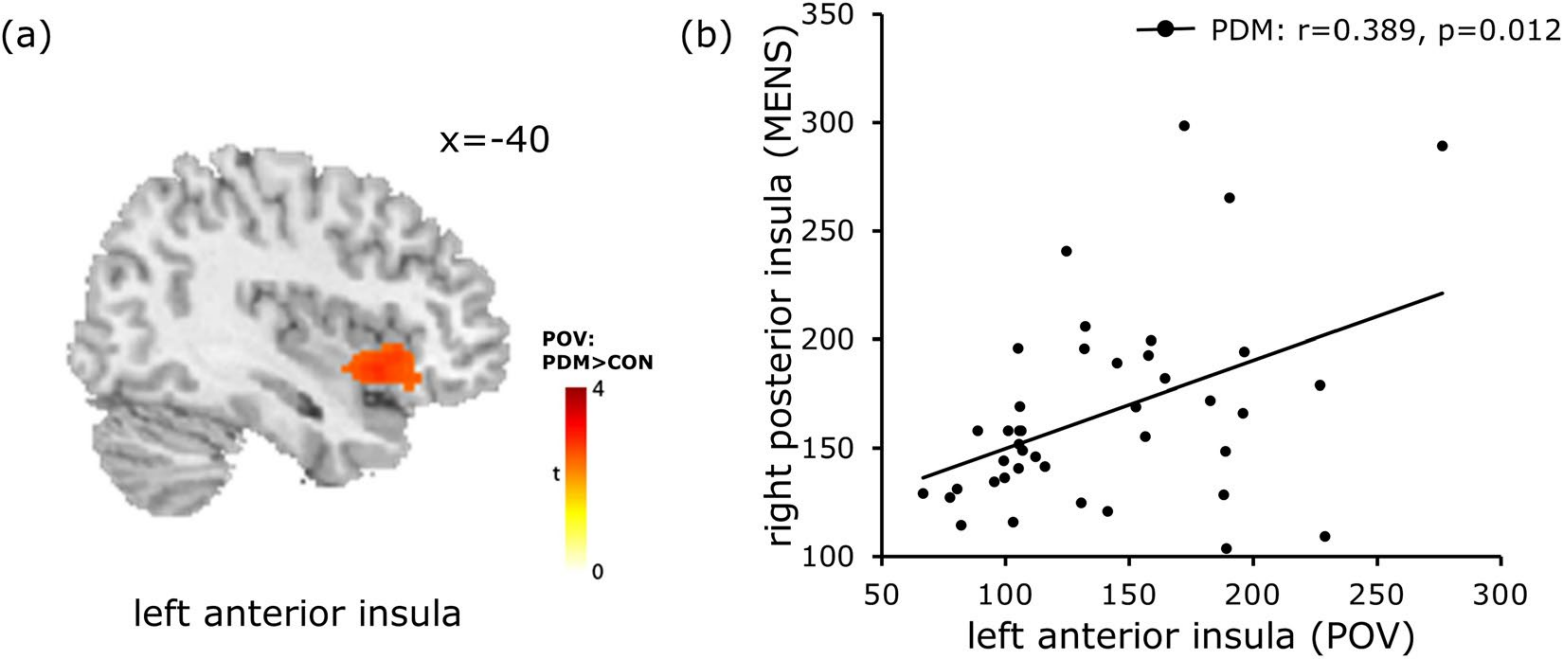
Cross-phase correlation of theta activity between the anterior insula (POV) and posterior insula (MENS) in PDM. Theta activity in the left anterior insula during the pain-free stage (POV) as shown in (**a**), is positively correlated with that of the right posterior insula during the painful stage (MENS), as shown in (**b**). POV, periovulatory; PDM, primary dysmenorrhea.

In the PDM group, as shown in Table 4, we observed a positive correlation between the right parahippocampal gyrus and recalled pain rating index assessed at the time of recruitment (*r* = 0.308, *p* = 0.038; Fig. 3a), which indicates that a relationship exists between cortical theta activity and pain experience. Moreover, our regression results revealed that MCC theta activity in the pain-free state (POV) could be a predictive factor for the severity of long-term pain at the sensory level (r = 0.325, p = 0.038; Fig. 3b). Finally, we observed that theta activity in the left anterior insula during the pain-free state (POV) was positively correlated with theta activity in the right pINS during the painful state (MENS) (Fig. 4).

**Table 4:** Results of correlation analysis between theta activity and psychological assessments

Phase	Anatomical label	Psychological assessments	Correlation, r-Value, *p*-Value	Permutation *p*-Value
MENS	Right parahippocampal gyrus	BDI	PDM: r = 0.327, p = 0.027*	0.025*
			CON: r = 0.002, p = 0.992	0.989
MENS	Right parahippocampal gyrus	Recalled PRI total values	PDM: r = 0.308, p = 0.038*	0.036*
MENS	Right posterior insula	STAI-S	PDM: r = 0.394, p = 0.007*	0.006*
			CON: r = 0.276, p = 0.063	0.064
POV	Left posterior middle cingulate gyrus	Recalled PRI-sensory	PDM: r = 0.325, p = 0.038*	0.018*
**Group**	**Anatomical label**	**Psychological assessments**	**Correlation** ***r*** **-Value**, ***p*** **-Value**	**Permutation** ***p*** **-Value**
PDM	Left pre/post-central gyrus	STAI-S	MENS: r = 0.376, p = 0.015*	0.014*
			POV: r = 0.037, p = 0.816	0.812

*p < 0.05. PDM, primary dysmenorrhea; CON, healthy controls; MENS, menstruation; POV, periovulatory; STAI-S, Spielberger State-Trait Anxiety Inventory-State; BDI, Beck Depression Inventory; PRI, Pain Ranking Index.

## Discussion

Theta oscillations in intrinsic brain networks may play a crucial role in the process of menstrual pain. In recent PDM studies based on resting-state fMRI (rsfMRI) data, we reported alterations in regional and cross-network functional connectivity associated with the pain modulation system^12,14^. In this study, we provide further MEG evidence of intra-network alterations and describe the dynamics of this process throughout the menstrual cycle. Our results revealed an increase of cortical theta activity in women with PDM and its association with the psychological factors pertaining to their pain experience (depression, state anxiety, and pain rating index) in several brain regions. Moreover, the effect size of comparison between MENS (pain) and POV (non-pain) phases in PDM group was smaller than that of comparison between PDM (pain) and CON (non-pain) groups in the MENS phase, demonstrating the effect of long-term menstrual pain on brain function. The parahippocampal gyrus may be involved in processing the experience of menstrual pain and encoding the associated psychological stress. The pINS processes the sensory information expressed in experiencing pain and the affective aspect of pain to the aINS. Finally, the hyperactive theta oscillations in the postcentral gyrus/SII of PDM subjects may underpin adaptations to sensory perceptions of pain. These findings suggest that the psychological outcomes of long-term pain experience would be manifested as theta oscillation in the sensory-limbic regions in young women with PDM.

In this study, we observed elevated theta activity in regions related to sensory and emotional processing, suggesting that theta oscillation may engage in both sensory and affective processing of pain. Theta oscillations are generally reported to relate to memory formation/integration, synaptic plasticity, and long-range synchronization^23^. The observed increase of theta activity in the parahippocampal gyrus, insula, anterior/middle cingulate, middle/inferior temporal gyrus in PDM subjects indicates its role in encoding of complicated perception and context related processes in experience of menstrual pain. Furthermore, we also found hemispheric lateralization of theta hyperactivity throughout the menstrual cycle. Theta hyperactivity was observed in the right hemispheric thalamo-cortical-limbic areas during periods of pain and the left hemispheric cingulate-frontal regions in both of painful and pain-free periods. These findings implicated that sensory functions of pain processing may be bilaterally engaged and emotional stress concurrent with menstrual pain was processed at the left hemisphere to prepare for the forthcoming event (menstrual pain) during pain-free stage.

Our results demonstrate that sub-regions of the insular cortex are involved in different processes of pain in women with PDM. The insula plays an important role as a multidimensional integration site for pain^24^. Previous studies have associated the pINS with the sensory-discriminative component (intensity, location, quality, and duration) and the aINS with the affective-motivational aspects pain and unpleasantness^25^. Our observation of increased theta activity in the pINS during the MENS is an indication of adaptive alterations in processing the sensory dimension of pain. Based on Mowrer’s two-factor model of fear conditioning^26,27^, we speculate that PDM subjects experience anxiety associated with pelvic cramping pain during the menstrual phase. It is also possible that these PDM subjects experience fear and anxiety even in the periovulatory phase, due to their previous experiences of pain returning every month. Our results showed correlations between anxiety and theta activity in the posterior insula during the painful phase (MENS phase) and that in the aINS during pain-free state (POV phase), respectively. These findings implicated that theta oscillation may encode information of unpleasant expression of menstrual pain and thus become as a trait-like functional alteration.

Our finding of a relationship between activity in the aINS and pINS across menstrual phases is consistent with our previous findings based on rsfMRI^12^. The aINS is a core region of salience network (SN) for salience information processing^28,29^. Increased connectivity between the aINS and default mode network (DMN) has been reported in patients with fibromyalgia^30^, which supports the view that the insula integrates pain information into higher-order cognitive conscious states of awareness. Craig *et al*.^31^ posited that the pINS receives interoceptive information of the entire body, which is then projected onto the aINS where it is used for the subjective evaluation of internal conditions. We speculate that the relationship between the aINS and pINS across menstrual phases may be contributed from pain-related sensory and affective information in the evaluation of interoceptive information in MENS phase and its projection to the evaluation of internal conditions during POV phase.

We observed an increase in theta activity in the left aMCC and pMCC of PDM subjects in painful as well as pain-free phases. Previous researchers have reported that the sub-regions of the cingulate cortex have played different roles in processes of painful stimulations^32^. Our findings of hyperactivity in the aMCC and pMCC suggest that PDM imposes adaptive changes in sensory-discriminative and cognitive-evaluative (attention) functions during pain processing. Based on a meta-analysis of pain imaging studies, the middle ACC (one of the cortical regions most frequently activated by pain), may reflect shifts in attention and be involved in monitoring the occurrence of sudden events^33^. Moreover, the middle ACC is part of the SN, which tracks how external stimuli capture attention^34^. In our recent PDM study based on rsfMRI data, we reported a decrease in the connectivity of left periaqueductal gray-seeded to the middle/rostral ACC (BA24)^14^. In our another rsfMRI study^12^, we identified hypoconnectivity between the vmPFC and dACC (DMN-SN network) in PDM subjects. In the present study based on resting-state MEG data, we further found the correlation between theta activity in the pMCC in a pain-free state with scores of the pain rating index, suggesting that resting pMCC theta activity during POV may be manifested as salience information about recent pain experiences.

Our results revealed that theta oscillations in the pre/post-central gyrus during the POV phase were higher than those observed during the MENS phase. As part of pain matrix, the pre/post-central gyrus is associated with sensorimotor processing and the sensory-discriminative aspect of pain experience. In a previous study, we reported on the hyperconnectivity of the dorsal attention network to circuits in the right postcentral gyrus during the MENS phase, which indicates that such changes could be adaptive in cases of PDM^12^. Wang *et al*.^35^ reported a significant increase in intrinsic regional homogeneity at the left postcentral gyrus in patients with idiopathic trigeminal neuralgia, which suggests that the postcentral gyrus is involved in modulating the sensory component of pain. Our finding of a positive correlation between state anxiety and theta activity in the left pre/post-central gyrus during the MENS phase provides further support for the Zhou’s hypothesis that changes in this area in response to pain would increase anxiety associated with the expectation of pain and thereby coordinate the processing of pain^36^. To summarize, our findings in this study suggest that the increase in theta activity in the pre/post-central gyrus in cases of PDM may be indicative of functional changes associated with adaptive sensory processing.

Individuals with PDM presented increased theta activity in the parahippocampal gyrus during the MENS phase, which was positively correlated with scores of depression. These findings are consistent with previous findings in which hyperresponsive activity was observed in the parahippocampal gyrus in stress-related populations, such as those with social anxiety disorder and posttraumatic stress disorder^37^. It has been proposed that the parahippocampal gyrus is involved in emotional processing and numerous cognitive processes, including episodic memory, contextual association, and stress-related disorders^38^. Experience of long-term menstrual pain is a stressful context, which could produce distressing memories for subjects suffering from PDM^39^. The correlation between theta activity in the parahippocampal and scores from depression and recalled pain rating indexes may be an indication of difficulty in coping with cyclic stressful events, resulting in the evocating negative emotions.

Our current findings reveal that the functional signatures of theta oscillations are a reflection of systematic alterations in the emotional, cognitive, and sensory components of pain processing. Our findings on theta oscillation could be applied to the treatment of PDM and chronic pain, via methods such as transcranial magnetic stimulation and transcranial direct current stimulation. Further cross-frequency analysis into thalamus-seeded functional connectivity with theta and gamma oscillations may be of considerable benefit to our understanding of the neural mechanisms underlying pain processing in women with PDM.

## Methods

### Subjects

All data in this study were obtained from participants in a previous PDM study who were eligible for neuroimaging studies^15^. We enrolled 46 females with PDM and 46 age-matched controls in this study. Subjects in the PDM group were 20–30 year old Taiwanese females with a regular menstrual cycle of approximately 27–32 days, a history of PDM longer than 6 months, cramping pain during the menstrual period in the previous 6 months with a rating exceeding 4 on a verbal numerical scale (VNS, 0 = not at all, 10 = the worst pain imaginable), as well as right-handedness confirmed by the Edinburgh Handedness Inventory. The healthy control females were selected to match the inclusion criteria for the PDM group with the exception that controls should have no pain during menses (VNS = 0). Exclusion criteria for all participants included a history of head injury, pathological pituitary gland disease, organic pelvic disease, psychiatric disorder, immediate plans for pregnancy or a positive pregnancy test, a history of childbirth, and having a metal/pacemaker implant. No subject had used oral contraceptives within 6 months or analgesics within 24 h prior to the initiation of the experiment. Subjects in the PDM group underwent pelvic ultrasonography to exclude cases of secondary dysmenorrhea caused by organic pelvic diseases, such as endometriosis or adenomyosis. All subjects were clinically examined and diagnosed in a gynecology clinic by a certified gynecologist. The study was conducted in accordance with the Declaration of Helsinki and was approved by the Institutional Review Board of Taipei Veterans General Hospital. Written informed consents were obtained from all participants prior to the experiment.

### Experimental design

MEG recordings and MRI scans were individually scheduled and acquired according to the commencement day of menstruation for each subject. All subjects received clinical and psychological assessments, MEG recordings and MRI scans at two time points during the menstrual cycle: the MENS phase (days 1–3 of the menstrual cycle) and the POV phase (days 12–16 of the menstrual cycle). Periovulatory phase was confirmed using a urinary luteinizing hormone test (Han Chiun Proper LH Rapid Test).

### Clinical and psychological assessments

The subjects in the PDM group completed the PCS^40^ and recalled their overall experience of menstrual pain using the McGill Pain Questionnaire (MPQ) during the initial interview. The women with PDM were then assessed with regard to their current experience of menstrual pain using the MPQ. All subjects underwent a psychological battery including State-Trait Anxiety Inventory (STAI), Beck Depression Inventory (BDI), and Beck Anxiety Inventory (BAI) during the MENS and POV phases.

### MEG/MRI data acquisition and preprocessing

From each participant, we recorded 3–5 min of MEG data with the subject in a eyes-open resting-state using a whole-head 306-channel neuromagnetometer (Vectorview^TM^, Elekta-Neuromag, Helsinki, Finland). MEG signals were sampled at 1001.6 Hz in conjunction with a 0.03–330 Hz band-pass filter. Three fiducial landmarks (nasion and bilateral preauricular points) and four head position indicator coils were used to locate the head of the subject relative to the sensor array using the Isotrak system (Polhemus Navigation Sciences, Colchester, Vermont, USA). The recorded signals were divided into 90–150 epochs of 2 sec each and processed using the signal space projection method for the removal of interference. Epochs exceeding 9000 fT/cm were considered artifacts and therefore rejected. Vertical and horizontal electrooculograms (EOGs) were recorded for use in identifying (for exclusion) all epochs contaminated by eye movements and/or blinks where the EOG exceeded 600 μV. These pre-processed data were then used for source analysis.

A 3 T Siemens Magnetom Tim Trio System (Siemens, Erlangen, Germany) was used to acquire T1 weighted anatomical brain MRI scans (MPRAGE, 192 sagittal slices, TR = 2530 ms, TE = 3.03 ms, TI = 1100 ms, flip angle = 7°, FOV = 224 mm × 256 mm × 192 mm, voxel size = 1 mm × 1 mm × 1 mm). Digitized fiducial points (nasion and bilateral pre-auricular points) and extra points on the surface of the head were used to align the coordinate frame of the MEG data and the anatomical MRI data. Co-registration of MEG data and anatomical MRI data was performed using an iterative closest point algorithm, an automatic alignment procedure included in the MNE software package^41^.

### MEG source analysis: Beamforming

The preprocessed MEG data were then filtered at 4–7 Hz (theta band) for each 2-sec epoch. Each signal was then analyzed using a beamforming method^42^, which yielded a spatial filter (with a unit-gain constraint) for each targeted brain location. These were used to minimize the variance imposed by filtering and to maximize contrast between filtered signals within the active-state period and control-state period. The control state was derived from 10 s of MEG recordings obtained in an empty room, and the active state was defined as a 2-sec epoch from the above noise-free MEG data. The forward solution was calculated using a homogeneous spherical conductor model. Tikhonov regularization^43^ was used to indicate the noise suppression factor. In cases where the noise level was high, the application of regularization was used to increase the stability of the solution; however, this resulted in a slight reduction in spatial resolution. We then constructed a theta activation map (a pseudo F-statistic map) by calculating the power ratio of the filtered theta activity between active and control states, and iteratively scanning the entire brain at an isotropic spatial resolution of 4 mm. For further statistical analysis, the activation map was spatially normalized to the Montreal Neurological Institute (MNI) space, in which the deformation field was estimated by spatially normalizing individual MRI to the MNI space using Brain Image Registration Tools^44^.

### Statistical analysis

Following the evaluation of variable distributions using the Shapiro–Wilk normality test, we employed the Mann Whitney U-test in the SPSS Statistics 20.0 package (SPSS Inc., Chicago, IL) to assess differences in demographic characteristics and psychological assessments between the PDM and CON groups. For each phase, theta activation maps of the two groups (PDM vs. CON) underwent whole-brain voxel-wise comparisons using the two-sample t-test module in SPM8, followed by cluster-based permutation tests to deal with the multiple comparison problem. The significance of the t-test was thresholded at an uncorrected voxel level p < 0.01 (cluster size k > 50 voxels). In permutations tests, data labels were shuffled and a distribution of mean theta activity for each surviving cluster, estimated based on 10,000 permutations, was used to test the significant value (p < 0.05). Furthermore, we also investigated brain changes in the PDM group between the MENS and POV phases using pair-t test. To examine the statistical power, we calculated the standardized effect sizes using Cohen’s d^22^.

Mean theta values of all the voxels within each surviving cluster obtained from between-group and between-phase comparisons were extracted for correlation analysis between brain activity and psychological scores using the Spearman correlation approach. Permutation test was further applied to deal with the multiple comparison problem. Linear regression analysis was also implemented to predict pain ratings of past and present menstrual pain experiences based on theta activity in the POV phase.

### Data Availability

All data generated or analyzed during this study are included in this published article.
